# Supplementary material for: Reduction spheroids preserve a uranium isotope record of the ancient deep continental biosphere
Source: Nat Commun. 2018 Oct 29;9:4505. doi: 10.1038/s41467-018-06974-9 (PMC6206012; doi:10.1038/s41467-018-06974-9)
Supplement: Supplementary file 1 — Supplementary Information [file 41467_2018_6974_MOESM1_ESM.pdf]

Reduction Spheroids Preserve a Uranium Isotope Record of the Ancient Deep Continental Biosphere

McMahon et al.

SUPPLEMENTARY INFORMATION

Supplementary Note 1: Uranium isotope systematics and enzymatic reduction

Uranium isotopes (U^{238}/U^{235}: $\delta^{238}U$) have been developed as a low-T paleo-redox proxy for the Earth's surface and upper crustal environments[1,2,3,4,5,6]. A thorough review of non-radiogenic U isotope fractionation has been recently published[7] but relevant material is summarized here.

The uranium isotope composition of crustal (and high-T) rocks shows a relatively narrow range of values averaging -0.29 ± 0.03‰ (ref. 8). In the modern Earth surface, biologically-mediated reduction of U in sediments under marine sub-oxic or euxinic bottom waters, or in pore fluids represents the biggest sink for uranium[2,5,9,10].

U isotope fractionation is thought to be controlled by the nuclear volume effect, with the most significant fractionation occurring during U(VI) reduction to U(IV), concentrating the heavy ^{238}U isotope in the reduced product[1,2,5,10,11]. Although theoretical predictions verified by chromatographic isotope-separation experiments suggest that redox equilibration should enrich U(IV) in ^{238}U abiotically[12,13], at low temperatures this effect has only been observed (so far) where reduction is biologically (enzymatically) mediated, probably for kinetic reasons[10]. Indeed, recent work[10] suggests that abiotic reduction by reduced mineral species and other natural reductants either does not fractionate or causes the opposite fractionation to biological reduction[1,10,14,15,16]. Based on this experimental evidence, ref. 10 suggested that U isotope fractionation in this way represented a proxy for biological reduction of uranium in natural low-temperature settings.

Isotopically heavy uranium is also found in magmatic titanite and zircon crystals as a consequence of high-temperature abiotic processes[17], and in hydrothermally altered ocean basalts and associated calcium carbonate veins, where it might reflect either biotic or abiotic fractionation processes[18]. However, the factors controlling uranium isotope fractionation in these systems are quite unlike those in the reduction spheroids analysed here. By contrast, sandstone-hosted roll front uranium ore deposits are genetically similar to reduction spheroids; both systems represent sharp redox boundaries occurring at low temperatures in the continental subsurface. It has recently been shown[19] that the uranium in roll front deposits is isotopically heavy as a consequence of enzymatic uranium reduction, validating the proposal of ref. 10 and demonstrating the feasibility of the results obtained in the present study.

Supplementary Note 2: Additional geochemical data

Carbon isotope data were measured at the Scottish Universities Environmental Research Centre. The (solid hydrocarbon) cores from spheroids at Dingwall yielded $\delta^{13}C_{PDB}$ values of -43.0, -44.0 and -44.0. Organic-rich cores from Heysham yielded values of -29.5 and -31.1. These values have been affected by ionizing irradiation[20,21,22] and in any case do not reflect the biogenicity of the spheroids themselves because the organic material pre-dates spheroid formation.

GC-MS analysis of the spheroid cores from Dingwall (**Supplementary Figure 1**) revealed the presence of hopane and sterane biomarkers consistent with biogenic hydrocarbons[22]. However, such biomarkers do not demonstrate that the reduction spheroids themselves were produced by microbial metal reduction, since the organic matter pre-dates the existence of the spheroids.

Activity ratios ($^{234}U/^{238}U$) are given in **Supplementary Table 1** and illustrated in **Supplementary Fig. 2**. Deviations from an activity ratio of 1 can be caused by incomplete digests (fractionating ^{234}U, but not ^{235}U or ^{238}U). Our digest method will not result in quantitative dissolution of all U bearing minerals (e.g., zircons). Alternatively, recent (<2Ma) interaction with oxidising or reducing fluids (e.g., groundwater) can also remove or add ^{234}U to sediments; the decay of this isotope causes alpha-recoil damage to the mineral lattice which allows ^{234}U to escape preferentially. Though many of our samples are within error of secular equilibrium, some cores of reduction spots are depleted in ^{234}U, and some halos are enriched. Overall, we observe no significant correlation between ($^{234}U/^{238}U$) and $\delta^{238}U$ (r^2 =0.39). Non-equilibrium values are observed most commonly in samples from Budleigh Salterton, but matrix samples from Budleigh Salterton do not show much deviation from secular equilibrium. This suggests a relatively closed system in which the very high concentration of uranium present in the cores promoted localised lattice damage and the leaching, diffusion or ejection of ^{234}U from the cores to the halos, but not beyond the halos (cf. ref. 20); such a process need not require recent interaction with groundwater and could perhaps be ancient. In any case, the abundance of uranium would minimise the effect of any recent overprinting on the overall $\delta^{238}U$ values, which we therefore consider most likely to record early diagenetic processes in the ancient deep biosphere.

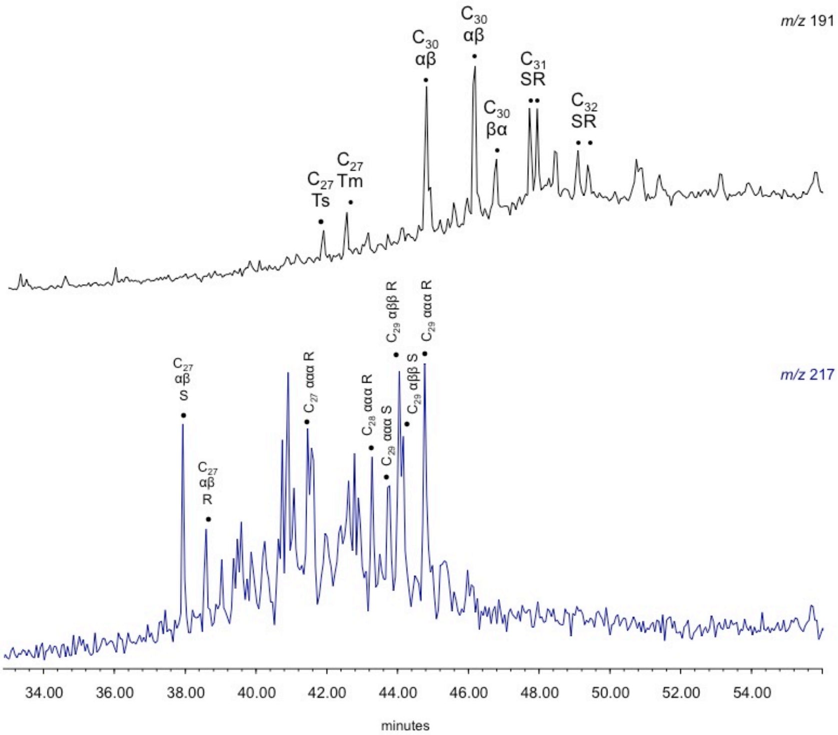

Supplementary Figure 1: Ion chromatograms (*m/z* 191). C_{27}Ts = C_{27} 18α (H)-22,29,30 trisnorneohopanes; C_{27}Tm = 17α(H)-22,29,30 trisnorhopane; C_{29} αβ = C_{29}17α (H),21β(H) hopane; C_{29} βα = C_{29}17β(H),21α(H) hopane; C_{31} αβ S = C_{31} 17α(H),21β(H) (22S) hopane; C_{31} αβ R = C_{31}17α(H),21β(H) (22R) hopane. (*m/z* 2217) C_{27} βαS dia = C_{27} 13β,17α(H) (20S) diasterane; C_{27} βαR dia = C_{27} 13β,17α(H) (20R) diasterane; C_{27} αααS = C_{27} 5α,14α,17α(H) (20S) sterane; C_{27} αααR = C_{27} 5α,14α,17α(H) (20R) sterane; C_{27} αββR + S = C_{27} 5α,14β,17β(H) (20S) + (20R) steranes.

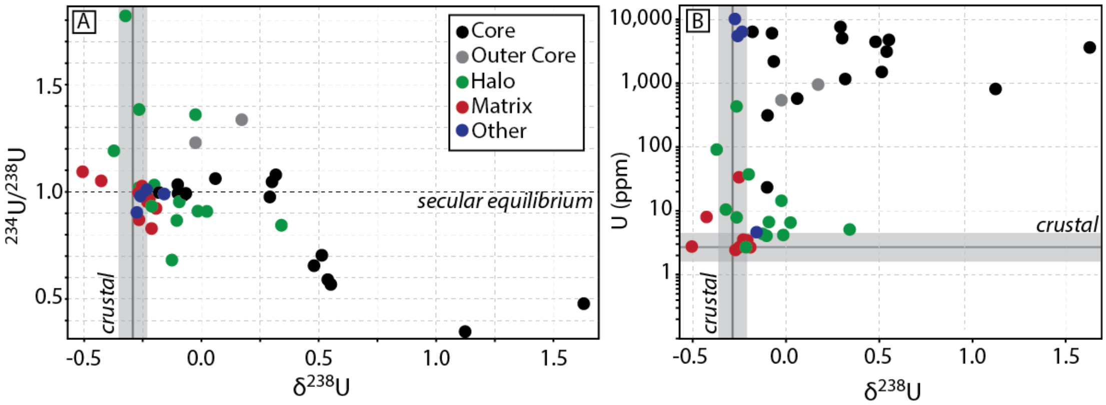

Supplementary Figure 2: A) ($^{234}U/^{238}U$) compared to uranium isotope ($\delta^{238}U$) composition of reduction spot samples and other samples. B) Uranium concentration compared to uranium isotope ($\delta^{238}U$) composition of reduction spot and other samples. There is no significant correlation between concentration and isotopic

composition of uranium, though cores tend to be enriched in both compared to crustal values. Matrix samples plot almost uniformly in crustal ranges of δ^{238}U (ref. 8) and are only moderately enriched in U concentrations (ref. 23).

Supplementary Table 1: Uranium isotope and trace metal data for reduction spheroids and other samples

Locality<br>Sample no.	Type	Organic fraction<br>(% loss)	Pb (206)<br>(ppm rock)*	Pb (208)<br>(ppm rock)*	Th<br>(ppm rock)*	U<br>(ppm rock)*	δ^{238}U<br>(‰)	δ^{238}U 2SE<br>(‰)	(^{234}U/^{238}U)	(^{234}U/^{238}U) 2SE
**BUDLEIGH SALTERTON**										
U1b	Core	9	514.7	434.2	8.91	1161.0	0.32	0.05	1.079	0.001
U1g	Halo	9	31.5	25.4	10.45	14.2	-0.03	0.05	1.360	0.001
U2b	Core	9	32259.6	27011.4	5.38	572.7	0.06	0.08	1.062	0.002
U2g	Halo	9	95.6	85.6	36.35	7.8	-0.27	0.04	1.384	0.0004
U3	Matrix	7	19.9	16.4	3.51	7.9	-0.43	0.09	1.051	0.002
BR-1	Matrix	5	27.2	25.8	8.22	2.3	-0.27	0.05	0.870	0.006
BR-2	Matrix	5	26.3	24.3	10.40	2.7	-0.51	0.07	1.093	0.003
BC1A	Core	3	663.7	575.2	13.15	811.2	1.12	0.05	0.348	0.003
BC0A	Outer Core	5	575.2	512.1	5.45	540.3	-0.03	0.04	1.229	0.002
BHA	Halo	5	22.1	17.4	9.50	10.4	-0.32	0.07	1.820	0.003
BC1B	Core	4	10574.5	9782.0	8.30	3628.4	1.63	0.04	0.479	0.003
BC0B	Outer Core	6	625.7	564.4	7.97	944.9	0.17	0.09	1.336	0.007
BHB	Halo	6	26.8	21.0	9.25	91.9	-0.37	0.04	1.191	0.00
**DINGWALL**										
U8a	Halo	7	11.5	9.0	8.85	36.9	-0.20	0.06	1.031	0.002
DG-1	Halo	7	9.8	8.6	6.25	5.0	0.34	0.05	0.844	0.008
DG-2	Halo	6	9.3	8.0	6.54	4.1	-0.02	0.09	0.910	0.007
DG-3	Halo	9	9.9	8.4	6.11	6.5	0.02	0.09	0.909	0.007
DR-1	Matrix	11	14.9	13.4	6.61	2.6	-0.19	0.04	0.923	0.006
DR-2	Matrix	6	18.4	16.7	10.78	3.5	-0.23	0.04	0.954	0.006
U9	Core	71	2491.7	1233.1	10.51	4456.0	0.48	0.03	0.656	0.001
U10	Core	76	1114.1	566.5	6.77	1499.7	0.51	0.05	0.704	0.001
DC-3	Core	40	1071.7	727.6	8.34	3130.6	0.54	0.05	0.590	0.006
DC-4	Core	45	3259.1	1475.0	10.04	5083.2	0.30	0.07	1.047	0.003
DC-6	Core	60	3452.6	2203.0	5.72	4780.7	0.55	0.06	0.569	0.006
DC-7	Core	67	5116.8	1020.5	11.99	7632.8	0.29	0.08	0.976	0.008
DW-B	Bitumen vein	94	3.1	2.6	0.55	4.6	-0.16	0.05	0.990	0.001
**HARTLEPOOL**										
U4c	Core	75	5614.4	4834.6	42.96	6179.2	-0.08	0.03	0.984	0.001
U4g	Halo	8	12.6	10.9	7.05	4.3	-0.13	0.05	0.682	0.001
U4R	Matrix	7	25.2	22.3	11.22	3.4	-0.21	0.08	0.829	0.002
**MILLPORT**										
U5	Core	9	41.0	34.1	8.33	23.1	-0.10	0.05	1.034	0.001
U6	Halo	12	39.8	24.1	2.16	431.2	-0.27	0.05	1.019	0.001
U7	Matrix	13	9.4	6.7	2.91	33.3	-0.25	0.05	1.027	0.001
**NIPIGON**										

SC	Core	8	45.7	21.2	8.33	311.8	-0.10	0.10	0.992	0.006
SH	Halo	10	8.8	7.3	10.28	6.6	-0.09	0.10	0.953	0.006
SR	Matrix	11	15.4	13.4	9.99	3.3	-0.22	0.03	0.966	0.001
**HEYSHAM**										
U11	Core	33	464.7	35.7	7.90	6399.4	-0.18	0.04	0.997	0.00
U12	Core	22	156.6	10.2	6.26	2189.1	-0.07	0.06	0.992	0.002
**BUNYEROO**										
8808-113	Matrix	4	10.0	9.7	23.42	2.4	-0.27	0.04	0.996	0.001
8808-111	Halo	5	6.5	6.2	20.70	2.7	-0.21	0.05	0.933	0.002
BG-IEH	Matrix	3	23.9	23.1	27.22	2.6	-0.25	0.10	1.022	0.003
"16-1"	Halo	3	6.2	4.9	6.73	4.0	-0.11	0.05	0.866	0.008
**HYDROTHERMAL VEINS**										
GOH-B	Great Ormes Head	52	462.6	171.1	5.51	5510.0	-0.26	0.07	0.980	0.003
U14	Laxey (Basement)	81	464.7	35.7	7.90	6399.4	-0.23	0.07	1.009	0.002
U16	Ord Burn (Granite)	87	3859.2	1428.4	6.38	10174.3	-0.28	0.08	0.904	0.004

*Derived from back-extrapolating the dissolved elemental concentration (in stock solution) to account for organic loss

Supplementary references

1. Stirling, C.H., Andersen, M.B., Potter, E.K. & Halliday, A.N. Low-temperature isotopic fractionation of uranium. *Earth and Planetary Science Letters* **264**, 208-225 (2007).

2. Weyer, S., Anbar, A.D., Gerdes, A., Gordon, G.W., Algeo, T.J. & Boyle, E.A. Natural fractionation of $^{238}U/^{235}U$. *Geochimica et Cosmochimica Acta* **72**, 345-359 (2008).

3. Montoya-Pino, C., Weyer, S., Anbar, A. D., Pross, J., Oschmann, W., van de Schootbrugge, B. & Arz, H. W. Global enhancement of ocean anoxia during oceanic anoxic event 2: A quantitative approach using U isotopes. *Geology* **38**, 315-318 (2010).

4. Wang, X., Johnson, T.M. & Lundstrom, C.C. Low temperature equilibrium isotope fractionation and isotope exchange kinetics between U (IV) and U (VI). *Geochimica et Cosmochimica Acta* **158**, 262-275 (2015).

5. Andersen, M.B., Romaniello, S., Vance, D., Little, S.H., Herdman, R. & Lyons, T.W. A modern framework for the interpretation of $^{238}U/^{235}U$ in studies of ancient ocean redox. *Earth and Planetary Science Letters* **400**, 184-194 (2014).

6. Andersen, M.B., Vance, D., Morford, J.L., Bura-Nakić, E., Breitenbach, S.F. & Och, L. Closing in on the marine $^{238}U/^{235}U$ budget. *Chemical Geology* ***420***, 11-22 (2016).

7. Andersen, M.B., Stirling, C.H. & Weyer, S. Uranium isotope fractionation. *Reviews in Mineralogy and Geochemistry* **82**, 799-850 (2017).

8. Tissot, F.L. & Dauphas, N. Uranium isotopic compositions of the crust and ocean: Age corrections, U budget and global extent of modern anoxia. *Geochimica et Cosmochimica Acta* **167**, 113-143 (2015).

9. Dunk, R.M., Mills, R.A. & Jenkins, W.J. A reevaluation of the oceanic uranium budget for the Holocene. *Chemical Geology* **190**, 45-67 (2002).

10. Stylo, M., et al. Uranium isotopes fingerprint biotic reduction. *Proceedings of the National Academy of Sciences* **112**, 5619-5624 (2015).

11. Schauble, E.A. Role of nuclear volume in driving equilibrium stable isotope fractionation of mercury, thallium, and other very heavy elements. *Geochimica et Cosmochimica Acta* **71**, 2170-2189 (2007).

12. Bigeleisen, J. Nuclear size and shape effects in chemical reactions. Isotope chemistry of the heavy elements. *Journal of the American Chemical Society* **118**, 3676-3680 (1996).

13. Fujii, Y., Higuchi, N., Haruno, Y., Nomura, M. & Suzuki, T., Temperature dependence of isotope effects in uranium chemical exchange reactions. *Journal of Nuclear Science and Technology* **43**, 400-406 (2006).

14. Rademacher, L.K., Lundstrom, C.C., Johnson, T.M., Sanford, R.A., Zhao, J. & Zhang, Z. Experimentally determined uranium isotope fractionation during reduction of hexavalent U by bacteria and zero valent iron. *Environmental Science & Technology* **40**, 6943-6948 (2006).

15. Basu, A., Sanford, R.A., Johnson, T.M., Lundstrom, C.C. & Löffler, F.E. Uranium isotopic fractionation factors during U (VI) reduction by bacterial isolates. *Geochimica et Cosmochimica Acta* **136**, 100-113 (2014).

16. Stirling, C.H., Andersen, M.B., Warthmann, R. & Halliday, A.N. Isotope fractionation of 238U and 235U during biologically-mediated uranium reduction. *Geochimica et Cosmochimica Acta* **163**, 200-218 (2015).

17. Hiess, J., Condon, D.J., McLean, N. & Noble, S.R. $^{238}U/^{235}U$ systematics in terrestrial uranium-bearing minerals. *Science* **335**, 1610-1614 (2012).

18. Noordmann, J., Weyer, S., Georg, R.B., Jöns, S. & Sharma, M. $^{238}U/^{235}U$ isotope ratios of crustal material, rivers and products of hydrothermal alteration: New insights on the oceanic U isotope mass balance. *Isotopes in Environmental and Health Studies* **52**, 141-163 (2016).

19. Bhattacharyya, A., Campbell, K.M., Kelly, S.D., Roebbert, Y., Weyer, S., Bernier-Latmani, R. & Borch, T. Biogenic non-crystalline U (IV) revealed as major component in uranium ore deposits. *Nature Communications*, **8**, 15538 (2017).

20. Hofmann, B.A. A uranium series disequilibrium investigation of reduction spheroids in red beds. *Schweizerische mineralogische und petrographische Mitteilungen* **71** (1991).

21. Hofmann, B.A. Organic matter associated with mineralized reduction spots in red beds. In: J. Parnell, H. Kucha, P. Landais (eds) Bitumens in ore deposits (Springer), 362-378 (1993).

22. Parnell, J. & Eakin, P. The replacement of sandstones by uraniferous hydrocarbons: significance for petroleum migration. *Mineralogical Magazine* **51**, 505-515 (1987).

23. Rudnick, R. L. & Gao, S. Composition of the continental crust. *Treatise on Geochemistry* **4**, 1-51 (2014).
